# Therapeutic options for patients with advanced atrial fibrillation: from lifestyle and medication to catheter and surgical ablation

**DOI:** 10.1007/s12471-020-01447-5

**Published:** 2020-08-11

**Authors:** L. Boersma, M. Rienstra, J. R. de Groot

**Affiliations:** 1 Department of Cardiology, St. Antonius Hospital, Nieuwegein, The Netherlands; 2 Department of Cardiology, Heart Center, Amsterdam Cardiovascular Sciences, Amsterdam UMC, University of Amsterdam, Amsterdam, The Netherlands; 3 Department of Cardiology, University Medical Center Groningen, University of Groningen, Groningen, The Netherlands

**Keywords:** Atrial fibrillation, Upstream therapy, Catheter ablation, Surgical ablation, Lifestyle

## Abstract

Atrial fibrillation (AF) is part of a vicious cycle that includes multiple cardiovascular risk factors and comorbidity which can promote atrial remodelling and AF progression. Most AF-related risk factors—hypertension, diabetes, sleep apnoea, obesity and sedentary lifestyle—are in essence modifiable which may prevent AF development. Treatment of associated cardiovascular conditions may prevent both symptoms and future cardiovascular events. For advanced forms of symptomatic AF refractory to lifestyle management and optimal medication, invasive ablation therapies have become a cornerstone. Although electrical trigger isolation from the pulmonary veins is reasonably effective and safe, more potent energy sources including high output-short duration radiofrequency, ultra-low cryo-energy, and electroporation, as well as more sophisticated arrays, balloons, and lattice-tipped catheter tools, are on their way to eliminate existing pitfalls and simplify the procedure. Electroanatomical navigation and mapping systems are becoming available to provide real-time information on ablation lesion quality and the critical pathways of AF in the individual patient to guide more extensive ablation strategies that may enhance long-term outcome for freedom of advanced AF. Surgical techniques, either stand-alone or concomitant to structural cardiac repair, hybrid, or convergent, with novel less invasive access options are developing and can be helpful in situations unsuitable for catheter ablation.

## Dutch contribution to the field

- Drugs and lifestyle studies with global impact from the Netherlands: RACE study programme from the Universities of Groningen (Prof I. van Gelder, Prof M. Rienstra) and Maastricht (Prof. H. Crijns).
- Catheter ablation studies with global impact from the Netherlands: Phased RF ablation studies (Prof L. Boersma).
- Surgical ablation studies with global impact from the Netherlands: FAST trial (Prof L. Boersma); AFACT trial (Prof J. De Groot).

## Introduction

Atrial fibrillation (AF) is a progressive disease, associated with increased morbidity and mortality [1]. Many factors contribute to the progression from infrequent paroxysms suppressible by drug therapy to prolonged symptomatic episodes that require chemical or electrical cardioversion, to chronic rhythm abnormality that may lead to exercise intolerance and heart failure. It is this more chronic state of prolonged arrhythmias that has been found most resistant to acute therapies such as antiarrhythmic drugs and invasive interventions. In this review we focus on the multifactorial approach that may be useful and needed to improve treatment outcomes.

## Lifestyle and comorbidity

Multiple cardiovascular risk factors are associated with the development and progression of AF. Atrial remodelling caused by these risk factors can promote AF and its recurrence. The majority of the AF-related risk factors—hypertension, diabetes, sleep apnoea, obesity, and sedentary lifestyle—are in essence modifiable and should be considered in the prevention and treatment of AF [2]. Treatment of associated cardiovascular conditions is of great importance since this treatment can reduce AF burden and symptomatology, but can also reduce the risk of future cardiovascular events [2]. This is in contrast to many other AF therapies that only have an effect on either AF burden or prognosis.

Hypertension is known to be the most common risk factor for AF. It has been reported that the prevalence of hypertension in AF ranges from 49–90% [3]. Even borderline hypertension is associated with increased risk for incident AF [4]. Optimal treatment of hypertension with angiotensin-converting enzyme inhibitors and angiotensin II receptor blockers has been shown to reduce the risk [5]. The RACE 3 trial showed that with targeted treatment of underlying risk factors of AF, such as reduction of blood pressure, maintenance of sinus rhythm is more successful [6].

Various studies have shown a strong association between obesity and AF [1]. A recent meta-analysis showed that patients with obesity have a 1.5 times higher risk than non-obese patients [7]. Furthermore, the observational LEGACY trial showed that long-term sustained weight loss reduced AF burden [8]. Moreover, moderate physical activity is also associated with a reduction in AF [9]. This can partly be explained by weight loss. However physical activity itself is also a contributing factor for incident AF. Physical inactivity is associated with a higher risk of cardiovascular events [10]. Improving physical activity may reduce that risk, in incident AF by up to 10% [11]. It should be noted that there is a U-shaped pattern between physical activity and risk of AF; extreme and prolonged exercise are considered to be a risk for AF [10, 12]. Offering patients the opportunity to participate in a cardiac rehabilitation programme might stimulate patients to be physically active and reduce the risk of AF recurrence [6].

Heart failure (HF) and AF often coexist sharing multiple risk factors. The epicardial adipose tissue may be one of the underlying mechanisms, as this local fat depot is enlarged in patients with a combination of HF and AF [13]. Furthermore, HF with preserved ejection fraction and AF share the same signs and symptoms. HF doubles the risk of incident AF. Treatment of HF may prevent AF and reduce the risk of AF recurrence. Treatment of HF with mineralocorticoid receptor antagonists is associated with a reduction in incident AF [14]. Moreover, it was associated with improvement of sinus rhythm maintenance in the RACE 3 trial, as well as in a meta-analysis of patients with AF in the presence or absence of HF [6, 15].

Another known risk factor for AF is obstructive sleep apnoea (OSA), which is often accompanied by obesity [16]. The estimated prevalence of OSA in AF patients is 50% [17]. Further, the risk of AF is twofold higher for patients with OSA compared with patients without OSA [18]. Treatment of OSA with a continuous positive airway pressure device (CPAP) has shown beneficial effects in recurrence of AF post-ablation [19]. Therefore screening for OSA in AF patients should be considered [2]. Since AF is a complex disease with multiple risk factors, comprehensive diagnosis and treatment of the underlying risk factors should be implemented in the daily clinical care of AF and should be part of the indication as well as the periprocedural management in patients scheduled for AF ablation.

## Catheter ablation for AF

Since the seminal publication by Haissaguerre et al.[20] in 1998, catheter ablation has become accepted therapy for AF refractory to conventional treatment. Electrical isolation of the pulmonary veins (PVI) was found to prevent AF by effective elimination of triggers, which remains the cornerstone for ablation treatment [21]. The strategy evolved from focal application of a radiofrequency current inside the veins, to segmental linear ablation, to wide area circular ablation, avoiding damage to the pulmonary veins while aiming at eliminating as much ectopy generating tissue as possible. Initially, freedom of AF was claimed in more than 90% of patients, and technical development led to electroanatomical navigation systems to guide ablation. As these procedures required expensive equipment and remained long and difficult, new catheter technologies were designed for quicker ‘single shot’ PVI. The phased radiofrequency multi-electrode array and the cryoballoon came onto the market[22, 23] and quickly became the most popular single short ablation strategies, which were as effective as existing single-tip ablation catheter procedures but with much shorter procedure times [24, 25]. The major adverse event rate during AF ablation procedures has become very low by increased operator skills, mapping and navigation systems, and optimising periprocedural anticoagulation, such that stroke, tamponade, severe groin bleeding and mortality have become rare [25]. However, during clinical follow-up and by using longer and more intense rhythm monitoring it has become apparent that recurrence of AF is much more frequent than reported in the early days. In the randomised FIRE&ICE trial comparing cryoballoon to irrigated radiofrequency single-tip ablation, freedom of AF after 18 months was less than 70%. The most common finding during redo-ablation remains recurrence of electrical conduction between the left atrium and one or more pulmonary veins, regardless of how extensively the index ablation was performed [26]. There is, however, still a potential for collateral damage to the oesophagus, the phrenic nerve, stroke due to char or thrombus embolisation, and perforation of the wall. This has urged improvements of existing technologies and strategies. Nowadays, single-tip radiofrequency catheters are irrigated and use contact-force sensing to perform safer, quicker, and more effective wide area ablation around the pulmonary vein, with improved AF freedom of up to 87% in low-risk patients with paroxysmal AF, if applications are done with optimal parameters and in close proximity to each other [27]. Several companies have developed new tools to create lesions with irrigated high-power output but short duration to retain safety and efficacy while further shortening the ablation time. New tools such as diamond-tip catheters allow similar quick radiofrequency delivery with rapid passive cooling by optimal heat exchange capacity, while a novel lattice-tip mesh-based catheter may allow more continuous lesion creation by conforming better to the tissue surface [28]. The latest cryoballoon iterations by Medtronic and Boston Scientific may provide more homogeneous circular freezing with improved and quicker isolation and higher reported freedom of AF outcomes. Still, the search for easier, safer, and quicker procedures continues and currently the field of AF ablation is exploding with novel tools and energy forms such as multi-electrode radiofrequency balloons and cryoballoon platforms from Biosense-Webster and Boston Scientific, or Adagio’s ultra-low temperature linear cryoablation with custom-built stylet driven shape optimisation for various ablation targets. One of the most promising new strategies is energy delivery by electroporation which is being developed by various companies. By delivering high voltage electrical field applications of only nanoseconds in the proximity of the tissue, the myocytes become irreversibly damaged without any effect on local temperature and specific to myocardial tissue, thereby avoiding char formation or insufficient energy transmission and collateral damage to surrounding structures. The next 5 years will reveal if all these new technologies can live up to their promise for improvement.

Aside from ablation technology, ablation strategy also needs improvement in those with non-paroxysmal AF or comorbidities with structural left atrial changes including dilatation and fibrosis, as PVI even with extensive left atrial substrate ablation shows freedom of AF in no more than half of the patients [29]. As the ablation strategy may require a more tailored individual approach, new strategies aim at electroanatomical substrate visualisation. High-resolution MRI may be used to determine left atrial wall fibrosis, which may be incorporated in the ablation strategy beyond PVI [30]. Several companies have developed systems for high-resolution mapping of electrical activation during AF to determine the individual AF mechanism to guide ablation. While TOPERA, the first in its kind, could not live up to initial high expectations (Brachmann et al. Heart Rhythm Society late-breaking clinical trial 2019; unpublished data), other technologies such as CardioInsight body surface mapping[31] and ACUTUS dipole density mapping[32] are still developing and testing sophisticated technologies and algorithms to characterise individualised AF patterns in real time to guide ablation and improve outcomes for those where PVI alone is not enough. New electroanatomical mapping technologies such as KODEX (Philips)[33] or working in an MRI instead of X‑ray imaging environment (Philips/Imricor) may further help guide and improve transmural lesion creation by real-time tissue evaluation.

## Advanced atrial fibrillation: treatment challenges

Whereas the preferred treatment strategy for patients with uncomplicated, symptomatic, paroxysmal AF has clearly become PVI via catheter ablation, the optimal strategy in patients with more advanced forms of AF, evident from (long-standing) persistent AF, a (severely) dilated left atrium or previously failed catheter ablations, remains incompletely elucidated.

In general, those patients are older than patients with paroxysmal AF and have more cardiovascular comorbidities, suggesting a more prominent place for upstream treatment as outlined above [34]. If patients with advanced AF are to be subjected to an invasive procedure, which approach is indicated and which additional ablation strategy should be added to the pulmonary vein isolation to restore sinus rhythm in this patient?

Before the emergence of catheter ablation, invasive treatment of AF was confined to surgical treatment. The Cox-Maze procedure, first described in 1987, underwent several modifications until the final lesion sets in the Cox-Maze 3 and 4 were defined [35]. Reported success rates were extremely high, but the procedure was associated with considerable morbidity and mortality, including an 8% pacemaker implantation rate [36]. More recent studies on the long-term follow-up, now including some form of rhythm monitoring, show that at 5 years of follow-up 59% of patients were in sinus rhythm (which is not equivalent to absence of AF recurrence during 5 years) [37]. A recent randomised trial demonstrated AF freedom in only 63% patients undergoing concomitant Maze surgery during mitral valve surgery, at the cost of more pacemaker implantations [38]. Thus, attempts were undertaken to combine the reported success rate of the Maze operation with a less invasive approach [39]. This procedure was further refined into a totally thoracoscopic procedure which, in a randomised comparison with standard of care catheter ablation in patients with mostly paroxysmal AF, yielded a higher efficacy at the cost of more procedural complications (mainly pneumothorax) [40, 41]. Recently, fewer complications have been reported [42]. A meta-analysis of all randomised treatment arms of catheter or thoracoscopic AF ablation studies suggests a higher efficacy of thoracoscopic ablation, but also again a slightly higher rate of procedural complications [43].

Recurrences of atrial arrhythmias frequently consist of atrial tachycardias, raising the question if transmural lines can be created reliably using a thoracoscopic approach on a beating heart [44]. Several approaches were developed to tackle this issue. Hybrid approaches complementing the thoracoscopic procedure with a catheter-based endocardial or epicardial mapping and ‘touch-up’ approach have been described in a single procedure setting, as well as in a staged setting [45–47]. Non-randomised data suggest that this approach is more efficacious than catheter ablation for persistent AF, and a randomised study on this issue is ongoing [48].

The autonomic nervous system plays an important role in the development of AF, and may be an attractive ablation target [49]. Indeed, ablation of the ganglion plexi added to PVI in patients with paroxysmal AF, was more efficacious than either of the two strategies in isolation [50]. However, it could not be excluded that the effect was due to more rigorous ablation around the pulmonary veins. The AFACT trial, randomising patients to a thoracoscopic ablation with or without ablation of the ganglion plexus, demonstrated that additional ganglion ablation with radiofrequency energy does not improve the results of the procedure but, to the contrary, is associated with more procedural complications [51]. Indeed, as mentioned above, the STAR-AF2 trial demonstrated no added value of several additional ablation strategies added to PVI in patients with persistent AF [29]. This leaves us with the question what the optimal approach is towards patients with advanced AF. One strategy to tackle this issue is to define an optimal ablation approach in these patients. Therefore, the multicentre APPROACH-AF study is now randomising patients with persistent atrial fibrillation between PVI only using state-of-the-art catheter ablation or thoracoscopic ablation and will provide data on the optimal ablation platform in those patients. A second approach is to define the optimal patient for invasive procedures. This requires understanding of the molecular substrate of persistent AF, and the recognition that ablation in some patients may just be futile. The PREDICT-AF study will provide insight into the molecular fingerprint of patients developing AF, and those who will remain free of AF [52].

We should acknowledge that there still is much we do not know about AF, its mechanisms, clinical implications, and optimal treatment. Although it may appear as only an electrical disorder that causes annoying symptoms, it is often facilitated and enhanced by many underlying pathophysiological mechanisms and disease states related to morbidity and mortality [1]. Although such underlying disease states may not preclude positive effects of invasive AF treatment, so far there is no hard evidence to support a reduction in mortality [53, 54]. Achieving absolute freedom of AF therefore remains a quest for the Holy Grail in decades to come.
